# The Design and Development of Woven Textile Solar Panels

**DOI:** 10.3390/ma16114129

**Published:** 2023-06-01

**Authors:** Neranga Abeywickrama, Matholo Kgatuke, Kalana Marasinghe, Mohamad Nour Nashed, Carlos Oliveira, Arash M. Shahidi, Tilak Dias, Theodore Hughes-Riley

**Affiliations:** Advanced Textiles Research Group, Nottingham School of Art & Design, Nottingham Trent University, Bonington Building, Dryden Street, Nottingham NG1 4GG, UKmatholo.kgatuke2009@my.ntu.ac.uk (M.K.); kalana.marasinghe@ntu.ac.uk (K.M.); jose.oliveira@ntu.ac.uk (C.O.); arash.shahidi@ntu.ac.uk (A.M.S.); tilak.dias@ntu.ac.uk (T.D.)

**Keywords:** electronic textiles, E-textiles, electronic yarn, E-yarn, smart textiles, solar, photovoltaic, energy harvesting

## Abstract

Over the past few years, alternative power supplies to either supplement or replace batteries for electronic textile and wearable applications have been sought, with the development of wearable solar energy harvesting systems gaining significant interest. In a previous publication the authors reported a novel concept to craft a yarn capable of harvesting solar energy by embedding miniature solar cells within the fibers of a yarn (solar electronic yarns). The aim of this publication is to report the development of a large-area textile solar panel. This study first characterized the solar electronic yarns, and then analyzed the solar electronic yarns once woven into double cloth woven textiles; as part of this study, the effect of different numbers of covering warp yarns on the performance of the embedded solar cells was explored. Finally, a larger woven textile solar panel (510 mm × 270 mm) was constructed and tested under different light intensities. It was observed that a P_MAX_ = 335.3 ± 22.4 mW of energy could be harvested on a sunny day (under 99,000 lux lighting conditions).

## 1. Introduction

This work focusses on the design and development of large area woven textile solar panels (building on previous work into the creation of solar electronic yarns [1]). The impetus of the work was to create a textile solar panel with normal textile properties (i.e., soft and drapeable), capable of harvesting a significant amount of energy (0.5 W under 1 Sun was targeted). This publication focusses on some of the design considerations and challenges in creating a woven textile solar panel of this type and presents important information about the effect of the woven structure on the performance of textile-embedded solar cells.

There has long been an interest in the creation of textile-based energy harvesting systems for both the powering of electronic textile (E-textile) devices as well as to provide a portable power supply for other wearables or mobile devices. Various E-textile-based energy harvesting techniques have been explored, such as triboelectric generators [2,3,4], piezoelectric generators [5], thermal energy harvesters [6], and electromagnetic induction-based energy harvesters [7]. While some of these techniques can generate significant power outputs (for example, triboelectric generators have been presented with power densities exceeding 500 Wm^−2^ [2]) or good textile properties (for example, the generator proposed by Xiong et al. looks like a normal textile and is highly deformable [3]), the continued development of solar energy harvesting textiles has remained popular.

Textile solar panels can be created using a variety of technologies resulting in textile solar panels with different power generating capabilities, stabilities, and textile properties (as discussed in detail in a recent review article [8]). Some of these fabrication methods have employed weaving to create final textile solar panels where 1D PV devices have been woven to create textile solar panels [9,10,11,12,13]; however, in these cases, limited details of how the textiles were woven are given, beyond what can be inferred from the photos. In all of these cases, while a porous structure is realized, the textiles do not have the appearance of a conventional textile. Weaving can also be used to create functional solar panels from separate PV fibers or tapes. In these cases, functional solar panels are often realized through the interlacing of electrodes and counter-electrodes (created through the weave). Some examples of this type of textile solar panel lack a normal textile appearance [14,15], or will lose the inherent porosity of the woven structure at the point where the electrolyte and a sealant layer is applied (for example [16]). Despite this, there are examples of textile solar panels with a normal appearance and softness through the use of conventional textile yarns in addition to the conductive fiber needed to create a solar energy harvesting system (i.e., the photoanode and counter-electrode) [17,18,19].

The work of Zhang et al. [18] and Chen et al. [20] discusses the effects of the chosen weaving pattern on the photovoltaic performance of the textile solar panel. Zhang et al. explored seven weave designs (single cloth) observing that a plain weave provided the greatest photovoltaic performance due to the larger effective illuminated area reaching the photoanode. The conversion efficiency was recorded as 0.8% for the plain weave sample, and 0.6% for the 8/3 stain weave structure (which gave the poorest performance) [16]. Chen et al. explored three weave designs (plain, twill, and satin) and made a similar observation, with the plain weave design leading to the greatest current density [18]. In both cases the plain weave resulted in the smallest number of fibers covering the photoanode, implying that the coverage of yarns was the greatest influencing factor.

Most textile solar panels presented in the literature (other than those created by affixing commercial panels onto the surface of a textile) are small (on the order of 50 mm × 50 mm or smaller is common), with one of the larger examples being a panel woven using PV tapes by Krebs and Hösel which was 250 mm × 250 mm [21] (however, in this case, the PV tapes where continuous and reasonably thick, affecting both the drapability of the textile and its appearance).

Textile solar panels can have many applications including for tents [22], bags [23], and outwear [24]. This has resulted in some commercial solar E-textile products in these areas [22,23,24]; however, in these cases flexible cells have typically been mounted onto the surface of the textile. This results in a product that lacks the normal textile feel, properties, and appearance where the cells are located. With the growing use of portable electronic devices in recent years, and an increasing interest in wearable E-textile devices, a comfortable, wearable power solution is desirable. With an E-textile solution that does not impair user comfort (i.e., normal breathability, etc.) garments that can be worn close to the skin that can harvest energy can be realized, such as T-shirts.

To date, very few existing textile solar panels possess many fundamental textile properties desirable for wearable applications [8]. One method of achieving a textile solar panel with good textile properties is through the use of solar electronic yarns, which can lead to a panel with a textile appearance and softness [1]. This is achieved by incorporating miniature solar cells in a discontinuous fashion at a yarn level and then using these solar electronic yarns (E-yarns) to weave panels that are drapeable, possess good shear behavior, and are porous. These solar E-yarns can be integrated into textile panels using existing industrial textile manufacturing techniques; in this work a hand-operated, computer-controlled Jacquard weaving loom was utilized.

This work provides a holistic account of the design and construction of a (relatively) large, woven, textile solar panel created using E-yarn technology. Solar E-yarns are created by soldering a miniature solar cell onto thin wires (such as Litz wires). Here c-Si solar cells were used as this is a mature technology with high power conversion compared to many emerging solar technologies, and c-Si cells are easy to source commercially. The soldered component is then encapsulated along with one or more supporting fibers within a solid resin micro-pod that is slightly longer than the length of the cell. This micro-pod protects the solar cell from mechanical and chemical stresses, while the supporting yarn helps support the wires and creates long continuous yarns (which are needed for further processing on industrial machinery): this ensemble is called the solar electronic filament (solar E-filament). The solar E-filament is then covered in fibers using knit braiding or conventional braiding to consolidate the structure and add strength, forming the final solar E-yarn. This paper first presents and characterizes a refined solar E-yarn design which was informed by other E-yarn studies (primarily [25,26]) and practical considerations regarding the solar cell dimensions. Previous solar E-yarn designs have proven to be highly mechanically robust and can survive multiple machine-washing cycles [1].

Textile swatches woven with solar E-filaments and solar E-yarns were then explored where different levels of fiber coverage over the solar element have been used. In the literature, it has been shown (as discussed above) that weave structures where fibers cover the smallest amount of the solar energy harvesting element obtain the best results (i.e., greatest energy capture). Here, this was explored in depth with four different levels of coverage using a double cloth structure. The focus on double cloth structures was design-informed as this structure gave the final textile a more consistent appearance. This was due to the different densities of warp yarns and the weft yarns in the weave. The textile solar panels utilized polyester for the majority of their construction given its robust nature, common use in technical textiles, and because it was previously used to cover solar E-filaments in earlier work [1,27,28]. The use of the same material for both the E-yarn covering and weave was preferred as ultimately this would improve the recyclability of textile solar panels at the end of their lives.

Solar E-filaments with multiple solar cells were used to construct a large area woven textile solar panel (including 1200 miniature solar cells) which was characterized under five different illumination conditions by taking measurements under direct sunlight on different days (an average of 27,800–99,000 lux was covered; approximately 0.23–0.83 Sun, assuming that 1 Sun is 120,000 lux). This work has been design driven, with the need to create a (relatively) large panel with good textile properties being the primary drivers. The work therefore also discusses some of the challenges in constructing and testing such a panel, as it is believed that this information will be useful to the research and development community.

## 2. Materials and Methods

### 2.1. Solar Electronic Yarn Design and Construction

The solar electronic yarns used in this work utilized a revised design to those employed in earlier articles [1,27] and shared similarities to a design presented by [28]. The choice for the revised design (compared to [1]) considered a handful of factors: The earlier use of non-insulated wires imposed restrictions on the spacing between solar cells (as larger gaps would allow the wires to potentially touch, creating a short circuit) and could result in failure in the presence of conductive liquids (such as sweat [25]). Another consideration was the solar cell size. By using insulated wires, the solar cells could be positioned further apart, so longer cells could be considered, and by using relatively large gaps between the cells, the final textile structure would still be deformable. It was desirable to minimize the number of solar cells used, to both simplify the construction of the final panel and minimize the potential points of failure; therefore, longer cells were used in this work (~5 mm long as opposed to ~3 mm long).

**Figure 1:**
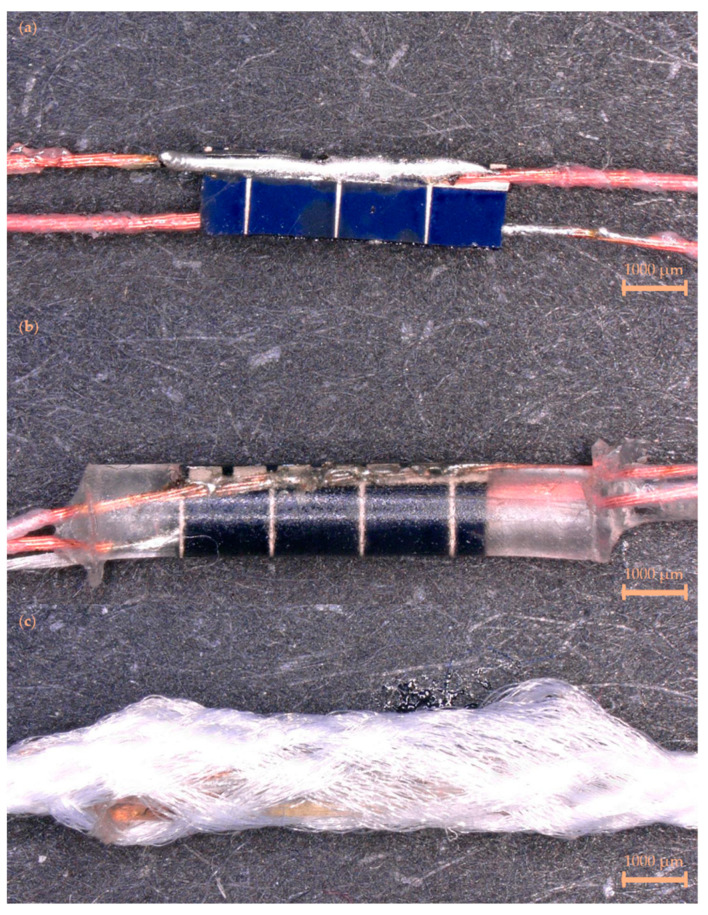
Stages in the construction of the solar electronic yarns. (**a**) A solar cell soldered onto two Litz wires. (**b**) A soldered solar cell encapsulated within a polymer resin micro-pod along with a supporting Vectran yarn (solar E-filament). (**c**) The soldered solar cell, Litz wires, and micro-pod were covered by a braided covering (creating a solar E-yarn; maximum outer diameter = 2.2 mm). The scale bars represent 1000 µm.

The solar E-filaments had two main production stages: First, two very flexible, commercially available, Litz wires composed of seven twisted enameled copper wires covered in a nylon covering (outer diameter = 254 µm; BXL2001, OSCO Ltd., Milton Keynes, UK) had a 5 mm long section of the nylon covering and enamel coating removed using a CO_2_ laser cutter (Orion Motor Tech 40 W CO_2_ Laser Engraver Cutter; Orion Motor Tech., PRC). The implementation of the laser was beneficial for process control as exact lengths of wire could be stripped at precise distances, resulting in correctly spaced solar cells when multiple cells were used. This specific Litz wire was used as it had been successfully implemented in other E-yarn devices (including solar E-yarns [28]) in past work. The cleaned wires then had a small amount of lead-free solder applied to them (diameter = 1.27 mm; comprising tin, natural rosin, silver, and copper; SA305; RS Components Ltd., Corby, UK) using a soldering iron (Antex XS25; Antex Electronics Limited, Plymouth, UK). One wire was then attached to a silicone mold and a custom silicon solar cell (active area = 4.34 ± 0.55 mm^2^; provided and cut by OPES Solutions, Hong Kong, PRC) was positioned under the wire with its large back electrode facing upwards. The back electrode was subsequently soldered onto the wire using the soldering iron. For solar E-filaments/solar E-yarns incorporating multiple solar cells, this process was repeated. The soldered solar cell(s) was subsequently inverted, and a second wire was placed atop the mold aligned with the top electrode of the solar cells. This was subsequently soldered using the soldering iron (see Figure 1a). The soldered solar cells were then fed into a discreate tubular mold (i.d. = 1.5 mm, length = 8 mm; silicone). The solar cell was positioned roughly halfway up the mold and a single multistrand Vectran yarn (Vectran™, Kuraray America Inc., Houston, TX, USA) was fed into the mold on the underside (opposite side to the photoactive area) of the cell. The mold was filled with an ultra-violet (UV) curable Acrylated Urethane resin (Dymax 9001-E-V3.5; Dymax Corporation, Torrington, CT, USA) and cured using a UV source (Dymax BlueWave^®^ QX4™; Dymax Corporation, Torrington, CT, USA); see Figure 1b. Again, this process was repeated for solar E-filaments/solar E-yarns with multiple solar cells.

Solar E-filaments could be converted into solar E-yarns through the use of a suture braiding machine (RU1/24-80, Herzog GmbH, Oldenburg, Germany) which constructed a fibrous sheath around the solar E-filament from 12 polyester yarns (48 f/167 dtex, no twist, 1 end; Ashworth and Sons, Cheshire, UK) consolidating the structure and strengthening the ensemble (Figure 1c). A lay length of 7 mm was used.

### 2.2. Weaving and Module Construction

For all of the woven textile samples presented in this work a double cloth woven structure was used. Plain weave structures were used for each cloth to create tubes into which the E-yarns were woven. Textile swatches containing single solar cells (in E-filament or E-yarn form), and a large woven panel comprising solar E-filaments with multiple solar cells per yarn (eight cells per yarn, 1200 solar cells total) were produced using a hand-operated, computer-controlled Jacquard weaving loom (Thread Controller 2, Digital Weaving Norway, Moss, Norway). A polyester warp (Ne 2/30; John L Brierley Textiles Ltd., Huddersfield, UK) was used. Identical yarns for the weft were used when E-yarns were not present. In the case of the large panel which incorporated multiple E-yarns, multiple weft yarns also separated each solar E-yarn in the main body of the panel. In all cases the intension was to orientate the solar E-yarns with the photoactive area facing upwards within the panel, however practically this proved to be difficult.

**Figure 2:**
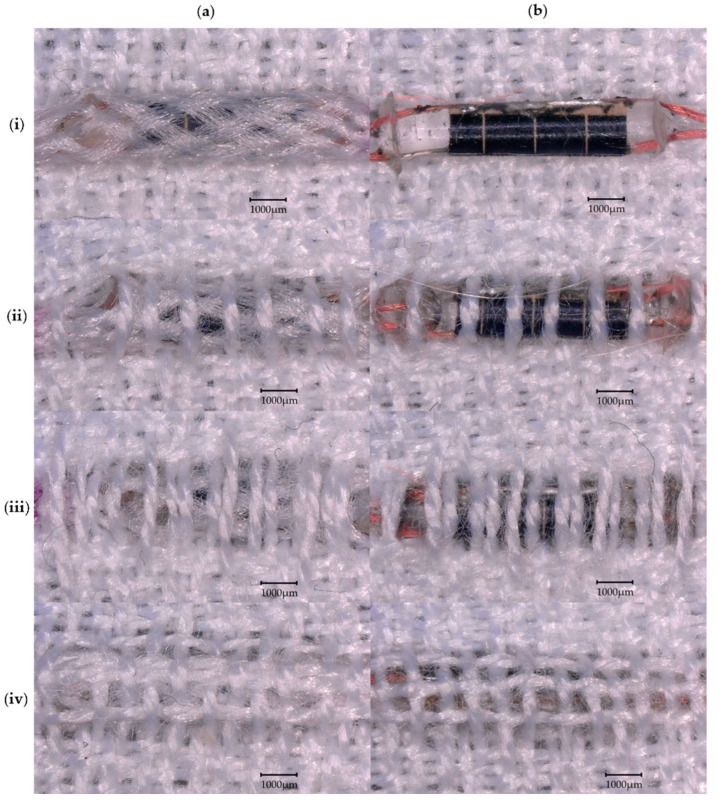
Microscope images (30× magnification) of the four different designs of textile swatches incorporating the solar E-yarns and solar E-filaments explored in this work. A double cloth woven structure was used with different quantities of yarns covering the solar cell element of the solar E-filaments/solar E-yarns: (**i**) no covering; (**ii**) four covering warp yarns; (**iii**) eight covering warp yarns; and (**iv**) twelve covering warp yarns/fully covered. Both solar E-filaments and solar E-yarns were employed: (**a**) solar E-yarns and (**b**) solar E-filaments (no braided sheath). The scale bars represent 1000 µm.

Four different small-scale swatch designs were explored, with each swatch being woven four times. These sets of samples were produced with solar E-yarns (Figure 2a) and solar E-filaments (without the braided covering; Figure 2b). The four weave designs used a double cloth structure with different numbers of warp yarns covering the solar cells on the face (the side where the photoactive side of the solar cells were meant to be facing) of the cloth. These included no yarns (micro-pod section of the E-filament/E-yarn completely uncovered; Figure 2i), four yarns (Figure 2ii), eight yarns (Figure 2iii), and twelve yarns (full coverage; Figure 2iv). A repeat set of the twelve covering yarn/fully covered swatches were produced and tested, and in this case, the average results from eight samples containing E-filaments and six containing E-yarns (due to two breakages) were presented. It should be noted that for all of the samples, four weft yarns covered the solar cell element of the solar E-filament/solar E-yarns; however, these often deformed around the micro-pod. As such these yarns are only clearly visible in the images of the samples shown in Figure 2iv (full coverage) and not for the other samples.

**Figure 3:**
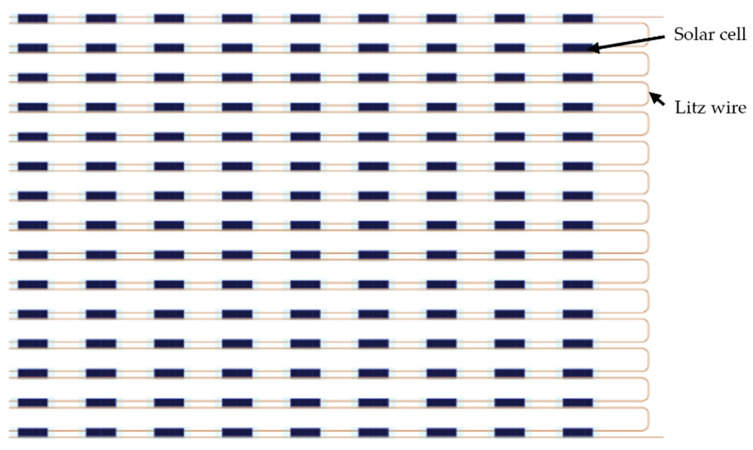
Schematic demonstrating how the solar cells comprising each textile solar panel module were networked together. Each solar E-filament was composed of eight solar cells soldered in parallel. The solar E-filaments were subsequently networked in series to complete a module.

For the large woven textile solar panel, 150 solar E-filaments were produced, each containing eight solar cells. These solar E-filaments were networked in series into modules incorporating 15 solar E-filaments each; this was done to increase the voltage output of the final panel, with a targeted voltage exceeding 5 V (the input for many portable devices). The module design is shown in Figure 3. Eight modules were produced within the textile and were networked together in parallel. The large area woven textile solar panel was constructed with solar E-filaments and a twelve covering yarn/fully covered double cloth woven structure. The rationale for this design choice is discussed in Section 3.3.

As both the warp and the majority of the weft yarns were comprised of polyester, other fibers were only present where a solar E-filament was used as a weft yarn. As stated earlier, the solar E-filaments contained a single Vectran yarn, and the Litz wires included only a small quantity of nylon. Therefore, the majority of the fibrous composition of the final textile solar panel was polyester.

### 2.3. Solar Electronic Yarn and Textile Solar Panel Testing

All solar cells soldered onto Litz wires, solar E-filaments, solar E-yarns, and solar E-filaments/solar E-yarns woven into small textile swatches were characterized using a benchtop solar simulator (LSH-7320 ABA LED solar simulator; Newport Corporation, Stratford, CT, USA). Measurements were taken using a benchtop digital multimeter (Keysight 34460A digital multimeter; Keysight Technologies, Santa Rosa, CA, USA). Open circuit voltage (V_OC_) experiments were conducted by directly measuring the voltage output of the device being tested; similarly, the short circuit current (I_SC_) was measured directly using the multimeter.

Given the lack of a large, uniform, consistent light source, the large woven textile solar panel had I-V characteristic curves recorded under direct sunlight. Light levels were measured using an RS 180-7133 portable light meter (RS Components Ltd., Corby, UK). As multiple (four) measurements were recorded at a given illumination level, the data is presented alongside the average illumination (light measurements were taken before and after the I-V curves were measured). Only datasets where all four measurements were taken sequentially have been presented. Most I-V characteristic curves used 21 resistance values (however, the 35,000 lux and 99,000 lux measurements lacked readings using 22.5 Ω, 51.1 Ω, and 67.6 Ω, which may have resulted in a slight underestimation of the maximum power achievable). Datapoints for each resistance value have been averaged, and P_MAX_ has been extracted from these averaged curves. As the measurements were taken outside of the laboratory, a portable multimeter (Mastech M-830B; Charlotte, NC, USA) was used to record these readings.

### 2.4. Microscopy and Measurements of Diamentions

All microscope images presented in this work were produced using a digital microscope (VHX-5000; Keyence, Milton Keynes, UK); this microscope was also used to measure the photoactive area of the solar cells employed in this study. The microscope had an in-built system for taking measurements optically.

Sample size measurements were taken by first allowing the fabric to relax, and the physical dimensions then being taken using a metal ruler.

### 2.5. Statistical Analysis

Unless otherwise stated, presented datapoints represent the average of four measurements. Error bars represent the standard deviation of the average to give an indication of the spread of the data. Error values presented throughout the manuscript are also given as the standard deviation. Data fittings were produced using Microsoft Excel (Version 2202, Building 14,931.20724; Microsoft Corporation, Redmond, WA, USA).

## 3. Results and Discussion

### 3.1. Solar Electronic Yarn Characterisation

The miniature solar cells displayed some variations in both their size and the photoactive area of the cell. After measuring the photoactive area of 55 solar cells using a microscope, the active area was observed to vary from 2.30 to 5.24 mm^2^, with an average active area of 4.34 ± 0.55 mm^2^. Miniature solar cells were soldered onto Litz wires and their V_OC_ and I_SC_ were recorded under 1 Sun intensity (see Appendix A.1). There was no clear functional relationship between the performance of the solar cells and the variations in the active area of the cell; therefore, it was believed that variations seen in the cell performance could be attributed to the quality of the solder joint achieved and potentially to manufacturing tolerances in the solar cells themselves.

**Figure 4:**
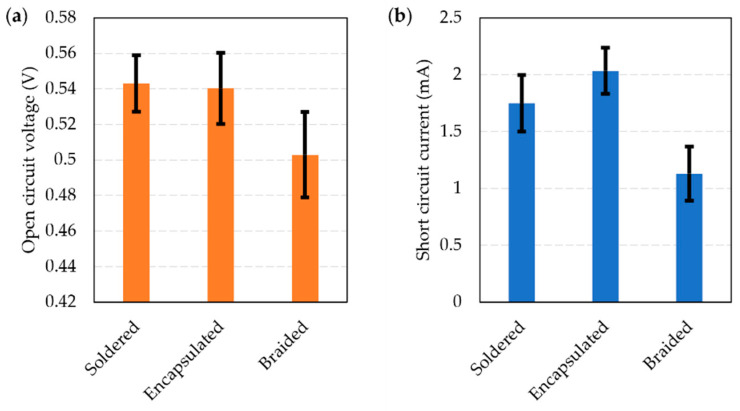
Performance of miniature solar cells soldered onto Litz wires and tested under 1 Sun illumination using a solar simulator. The figure shows the average performance with the cells just soldered onto Litz wires, encapsulated within a polymer resin micro-pod (solar E-filament), and when the ensemble was covered with a braided structure (solar E-yarn). (**a**) Open circuit voltage (V_OC_). (**b**) Short circuit current (I_SC_).

A set of 19 solar E-yarns (containing single solar cells) were prepared. Each solar E-yarn was tested at each stage of the manufacturing process to characterize their performance at each of these stages. While similar characterizations have been conducted in earlier work [1,28], this had used a different solar cell and slightly different solar E-yarn architecture, so a full understanding of the performance of the solar E-yarns used in this work was sought for completeness. Open circuit voltage (Figure 4a) and short circuit current (Figure 4b) are shown below.

There was a negligible change in the open circuit voltage between the soldered and encapsulated stages (V_OC_ = 0.543 ± 0.016 V and V_OC_ = 0.540 ± 0.020 V), with a slight reduction observed after the braiding stage (V_OC_ = 0.503 ± 0.024 V). A small increase in I_SC_ was observed between the soldered and encapsulated stages, with I_SC_ = 1.748 ± 0.247 mA increasing to I_SC_ = 2.033 ± 0.203 mA. A reduction to I_SC_ = 1.130 ± 0.238 mA was seen when the encapsulated solar cell was covered in a textile braid. This general trend of the I_SC_ increasing after the encapsulation stage and then dropping after being covered in a braided or knit braided fiber covering is in-line with previous work [1,28]. The increase in I_SC_ after encapsulation can be attributed to the lensing and light trapping effects of the micro-pod, while the reduction after being covered in a fibrous sheath (the braid) is likely due to light scattering and the absorption of light caused by the fibers [1,28].

The effect of the angle of incident light on the performance of the solar E-yarn was also studied using three solar E-yarns at each stage of the production process. As observed in previous work, the solar E-yarns performed best when the photoactive element directly faced the light source. Overall, the effect of angle on performance (I_SC_) was somewhat reduced at certain incident angles after the braiding step. Full details of these experiments are provided in Appendix A.2.

### 3.2. Solar Electronic Yarns Embedded within Woven Swatches

**Figure 5:**
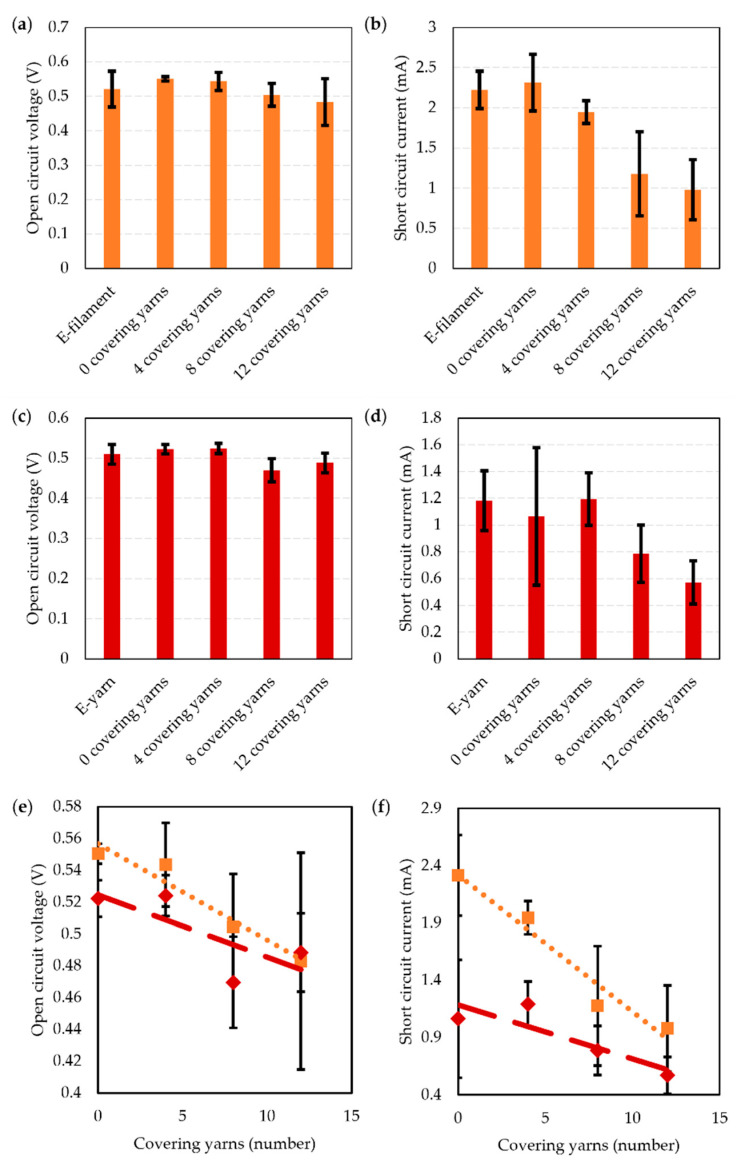
Performance of woven textile swatches incorporating solar E-filaments and solar E-yarns with a single embedded solar cell. A comparison of the performance prior to integration into a woven structure, and then with different levels of covering is presented. (**a**) E-filament open circuit voltage. (**b**) E-filament short circuit current. (**c**) E-yarn open circuit voltage. (**d**) E-yarn short circuit current. (**e**,**f**) Data has also been displayed comparing the woven samples with integrated solar E-filaments (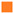, 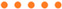) and solar E-yarns (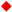, 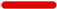) only as a function of covering yarns (re-using datapoints from a to d). (**e**) Open circuit voltage (V_OC_). (**f**) Short circuit current (I_SC_). The data fittings are provided below (in Table 1). For the data presented in this figure, each datapoint presents an average taken from four samples (except for when 12 covering yarns were employed where 8 E-filament samples were tested and 6 E-yarns samples where tested, or where the E-filaments or E-yarns were tested where 20 and 14 samples were tested, respectively).

Solar E-filaments and solar E-yarns incorporating a single solar cell were woven into textiles and characterized. Experiments were conducted to look at the different levels of covering over the solar cell elements in the woven structure so that a tradeoff between the solar panel’s technical performance and the textile appearance of the panel could be reached (Figure 5).

A linear relationship was observed between the number of covering warp yarns and the V_OC_ and I_SC_ for both the solar E-filaments and solar E-yarns. In both cases a small decrease in V_OC_ was observed with an increase in the number of covering yarns. Further, for both the solar E-filaments and solar E-yarns, the I_SC_ also reduced with the number of covering yarns; however, a significantly larger reduction in I_SC_ was seen when the solar E-filaments were tested. The V_OC_ and I_SC_ were similar for both the solar E-filaments and solar E-yarns when 12 covering yarns were used, which provided a good textile coverage over the photoactive area of the devices.

**Figure A2:**
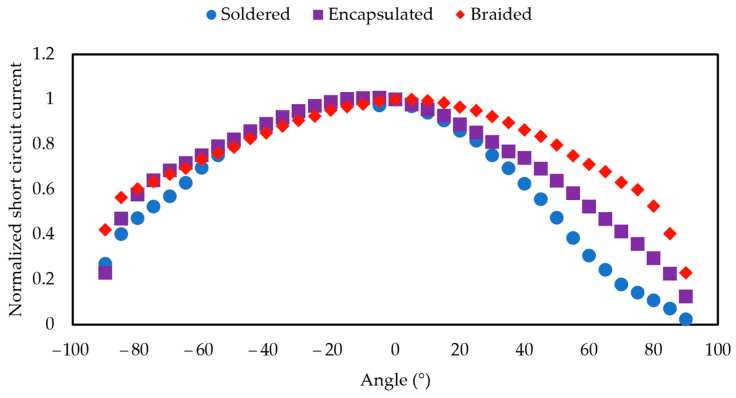
Normalized and averaged I_SC_ for three solar E-yarns when exposed to 1.0 Sun, with the light source at different incident angles. The figure shows the performance of the cells soldered onto Litz wires (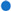), after the soldered solar cells were encapsulated within a polymer resin micro-pod (solar E-filament; 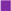), and when the ensemble was covered in a textile braid (solar E-yarn; 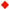).

There was a wide range of results collected between different swatch samples. This was not believed to be due to any intrinsic variability in the E-filaments/E-yarns, as normalizing the data to test results from the same solar E-filaments prior to weaving did not have a significant effect on this observed variability. Instead, it was believed that the variability was largely due to the difficulty in orienting the solar E-filaments/solar E-yarns within the woven structure so that the photoactive element faced upwards. Poor orientation of the embedded solar cell relative to the light source had the potential to significantly reduce the light reaching the cell (as seen in Figure A2). Orienting the embedded solar cell was particularly difficult when greater numbers of covering yarns were employed, as the cell could not be seen as easily during the weaving process; the results support this as greater variability was observed when eight and twelve covering yarns were employed. An alternative possibility is that the stresses of the weaving process damaged the solar E-filaments/solar E-yarns. At least two solar E-yarns were damaged and broke during the process of weaving test swatches; it is possible that further solar E-filaments/solar E-yarns were damaged but still functional.

The fitting and coefficient of determination from Figure 5 are shown in Table 1.

It can be observed from Table 1 that there is a strong correlation between the electrical parameters (V_OC_ or I_SC_) and the number of covering yarns when the solar E-filament was used, but a very poor relationship was seen when using the solar E-yarns (for V_OC_ R^2^ = 0.5736). This poor relationship is possibly a result of larger manufacturing tolerances when weaving the swatches containing solar E-yarns. As mentioned above, correctly orienting the embedded solar cell during the weaving process was difficult. Given the additional fiber layers, this was a greater challenge when the solar E-yarns were used.

The performance of the woven textile swatches as a function of the angle of the incident light was sought; however, preliminary results showed significant variations between the samples when tested. Specifically, the angle that provided the optimal I_SC_ was not at 0°, with the angle producing the optimal value varying between samples (for example, for the sample made using solar E-filaments and with four covering yarns in the weave, the optimal angle varied between +5° and −30°). This was likely due to variations in the orientations of the individual cells and the difficulty in correctly orientating the cells during the weaving process; supporting the earlier theory that this had led to variations in the results between samples as such further characterization experiments were not conducted.

### 3.3. Construction and Testing of the Large Woven Textile Solar Panel

The construction of the large woven textile solar panel required significant people, resources, and materials to actualize. To ensure that the final panel had a suitable appearance and feel, to understand the realistic challenges of creating a panel of this size, and to ensure that any final panel would produce the targeted 0.5 W under 1 Sun illumination, a series of test textiles were produced. This included the production of small textile swatches incorporating mostly dummy E-yarns (no solar elements were present, but the wires, micro-pods, and covering was the same); while mostly comprised of dummy E-yarns, these samples included a single functional solar E-yarn in the center of the sample. Multiple panels with around 300 solar cells within them (representing a panel that would generate approximately 0.125 W of power under 1 Sun) were also produced. These panels otherwise used the designs, materials, and processes described in the ‘Materials and Methods’ section for the large woven textile solar panel. One exception was that some of the test panels incorporated four solar cells per solar E-yarn, and not eight. Testing data for these samples has not been incorporated into this manuscript, as aspects of the data are not comparable (i.e., different testing methods were used); however, the knowledge established was useful in producing the final panel. The production of these panels also acted as a ‘trial run’ providing the opportunity to practice manufacturing steps and refine personal techniques.

Experiments conducted using the sample woven textile solar panels did inform the final testing process. Large solar simulators are expensive and relatively uncommon in an academic environment, making accessing one difficult. As such, alternative light sources, mainly a floodlight bank (HL 313.01 Artificial light source, Gunt, Hamburg, Germany) and a large LED light source (6F08 LED Floodlight, NIKEEYEN, PRC), were used for testing. In both cases the light sources did not provide a spectrum identical to that of sunlight, which resulted in the textile solar panels providing a very low output for the LED light source. In the case of the floodlight bank, the lights heated the panel significantly, which would affect the power output. The challenges with using these sources led to the decision to test the panel under natural sunlight.

The final woven textile solar panel was woven using solar E-filaments and with a woven structure where yarns fully covered the micro-pods. This did not provide the best trade-off between performance and appearance; it can be seen by comparing Figure 2 and Figure 5 that the solar E-yarn fully covered within the woven structure provided the best textile appearance and comparable performance (in terms of solar energy harvesting) as the equivalent panel that used solar E-filaments. The solar E-filaments were chosen as these were easier to orient during weaving (although this was still difficult). Removing the additional production step also simplified construction and removed a further possible point where the E-filaments could fail.

**Figure 6:**
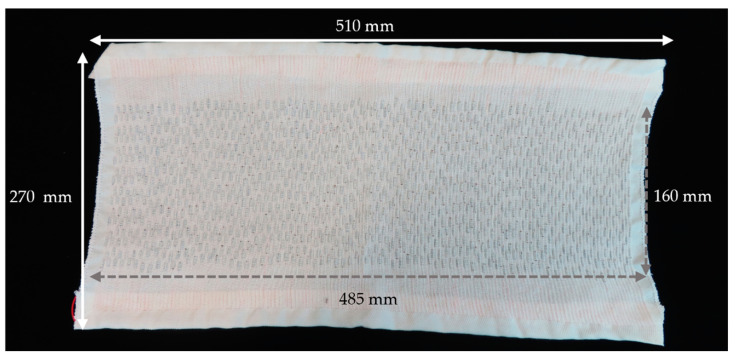
Image of the completed woven textile solar panel. The image has been annotated to show the approximate size of the overall textile as well as the size of the area that incorporates the miniature solar cells.

The completed woven textile solar panel, containing 1200 miniature solar cells, is shown in Figure 6.

**Figure 7:**
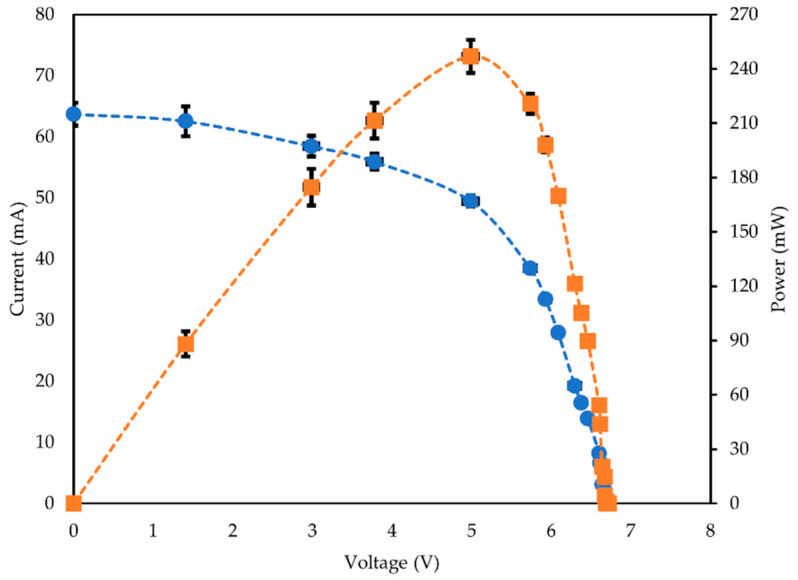
Characteristic current–voltage (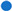) and power–voltage curves (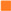) for the woven textile solar panel when tested outside under 81,000–83,000 lux lighting conditions (~0.7 Sun). P_MAX_ = 247 ± 9 mW. The data presented are averaged from four discrete datasets. The dashed lines have been included as a guide for the eye only.

**Figure A3:**
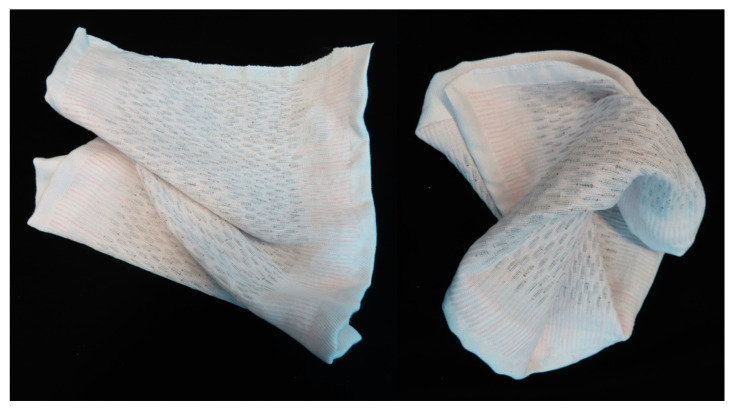
Two photographs of the large area woven textile solar panel after having undergone mechanical deformation.

The panel had normal textile properties and was highly deformable (see Figure A3). The completed panel was subsequently tested in direct sunlight for five different lighting conditions. Figure 7 shows characteristic current–voltage and power–voltage curves when the panel was tested under 82,000 lux lighting conditions. Full raw datasets for all of the lighting conditions (27,800 lux, 35,000 lux, 58,500 lux, 82,000 lux, and 99,000 lux) which the panel was tested under are available in the data archive associated with this article.

**Figure 8:**
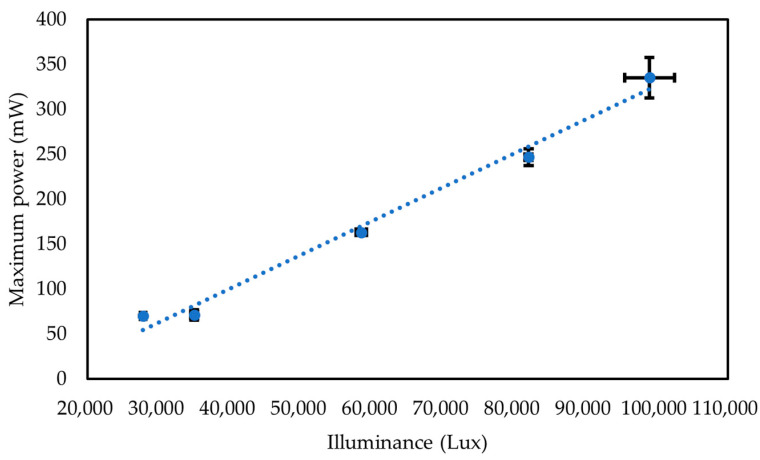
Performance of the woven textile solar panel under different lighting conditions. Each datapoint is averaged from four repeated sets of data. The data fitting is provided below. The error bars for the illuminance show the maximum and minimum illumination level recorded during the experiments.

P_MAX_ was extracted for each of the characteristic I-V curves and plotted against light intensity, as shown in Figure 8. This demonstrated the woven textile solar panel’s operation at different lighting levels.

As expected, the maximum achievable power was linearly related (R^2^ = 0.9868) to the illumination level with the relationship shown in Equation (1).

P_MAX_ = 0.0038I − 51.819 (1)

where P_MAX_ is the maximum power, and I is illuminance. The maximum power recorded was on a sunny day at 99,000 lux conditions, with P_MAX_ = 335.3 ± 22.4 mW. Characterizing the operation of the solar panel under different lighting conditions can allow for an understanding for how the panel would operate during different weather conditions (such as when it is overcast), at different times of the day (low light conditions), or when in shadow (which would occur sometimes on a worn garment as a wearer moves around). Figure 8 both proves that the panel can operate in these conditions, and also provides an indication of the expected power output in such scenarios.

## 4. Conclusions and Future Work

This work presented a relatively large woven textile solar panel that has been proven to harvest P_MAX_ = 335.3 ± 22.4 mW of energy under 99,000 lux lighting conditions (~0.83 Sun). The panel has normal textile properties, which was achieved by creating the panel by weaving a series of solar E-filaments together. While the panel generates significantly less power than a conventional panel of its size (largely due to the discontinuous nature of the solar elements), the reported output is still sufficient for powering many portable electronics or E-textile devices.

This work has focused on the effect of the level of covering over the photoactive component due to the weave design on the performance of the embedded solar element. It was observed that the weave design had a limited effect on the performance of solar E-yarns (that had a braided textile outer sheath) but that the level of yarn coverage did affect the performance of the solar E-filaments (that did not have a fibrous sheath covering). This was significant as this better represents many of the fiber- or yarn-based solar energy harvesting solutions from the literature, and this work may prove helpful to those innovations.

The relatively large (510 mm × 270 mm) woven textile solar panel produced in this work was also tested under different lighting conditions.

Further work will explore the potential of producing such a panel using automated (or semi-automated) processes as this will be necessary for producing this type of solar panel at scale. The process is currently labor-intensive, which would be the greatest cost if panels of this type were to be produced commercially. The researchers have previously developed a technique to encapsulate small-scale components using an automated system [29], so future work will focus on automating a process for soldering the small solar cells. Based on experience gained during this study, it is believed that advanced, multi-axis robots can be used to replicate the soldering process currently conducted by people. This process will first be refined using simpler components (i.e., SMD resistors) before being adopted for soldering solar cells.

While this work focused on a rectangular panel, in principal panels of different sizes and shapes could be produced depending on the intended application or location on a garment; for example, it may be desirable to integrate panels onto the shoulders of a garment. Ultimately, there will be design trade-offs between the density of the solar cells within the textile, cell size, power output, and the textile properties of the resultant panel. A comprehensive study of the density of the micro-pods on the key textile parameters of the panels should therefore be conducted so that these design rules can be understood.

Further work will also explore the durability of this type of panel and examine the repairability of the device. Earlier work has shown that the solar E-yarns are highly robust [1]; however, it is believed that the revised design shown in this work will be stronger, given the uses of Litz wire, therefore increasing the lifetime of the device. A full battery of material tests will be required to be able to fully understand the device’s potential lifetime. As the E-yarns are highly robust, it is expected that the yarns could be removed from solar garments following their end-of-life and reused; however, understanding the practical viability of this will require further research.

## Figures and Tables

**Table 1:** Open circuit voltage (V_OC_) and short circuit current (I_SC_) as a function of the number of warp yarns covering the surface of the photoactive area of the solar E-filaments or E-yarns (C_y_). These fitting parameters have been extracted from Figure 5.

	Data Fitting	Coefficient of Determination (R^2^)
Solar E-yarn (V_OC_)	V_OC_ = (−0.004)C_y_ + 0.5245	0.5736
Solar E-filament (V_OC_)	V_OC_ = (−0.006)C_y_ + 0.5566	0.9432
Solar E-yarn (I_SC_)	I_SC_ = (−0.0473)C_y_ + 1.1879	0.7597
Solar E-filament (I_SC_)	I_SC_ = (−0.1194)C_y_ + 2.3212	0.9544

**Figure A1:**
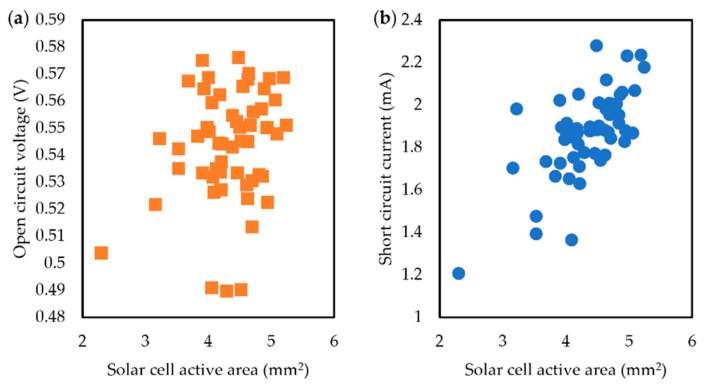
Characterization of miniature solar cells soldered onto Litz wires under 1 Sun illumination. (**a**) Open circuit voltage compared to the active area of the solar cell. (**b**) Short circuit current compared to the active area of the solar cell.

## Data Availability

The data generated and analyzed during this study are included in this article. Raw data files used to generate the figures shown in this work are available at https://doi.org/10.6084/m9.figshare.23267819.v1.

## Appendix A

### Appendix A.1. Soldered Solar Cell Performance as a Function of Active Area

As described above, the solar cells soldered onto Litz wires were characterized under 1.0 Sun illumination, as shown in Figure A1.

The cells produced an average I_SC_ of 1.87 ± 0.20 mA and V_OC_ of 0.54 ± 0.02 V. Figure A1 showed no clear relationship between the photoactive area of the soldered solar cells and I_SC_ or V_OC_. The variations observed between the soldered cells may be attributed to variations in the quality of the soldering or due to the manufacturing tolerances of the silicon solar cells. The photoactive area would also sometimes reduce slightly due to poor soldering, as solder could cover some of this area, which is not accounted for in the graph above. Regardless of the cause, the variations were observed to be minimal.

### Appendix A.2. Characterization of the Solar E-Yarns at Different Incident Angles of Light

As the solar E-yarn design in this work differed from that used in earlier work, the characterization of the solar E-yarns at different incident angles of light has been included for completeness (Figure A2).

Soldered solar cells, solar E-filaments, or solar E-yarns were positioned atop a rotatable platform that was fitted with a goniometer. The devices were positioned with the photoactive area of the solar cells facing the light source. The platform was rotated in 5° increments ±90° with the rotation taking place in the plane parallel to the long axis of the yarn: I_SC_ was recorded at each angle. As cell performance varied, and performance was different at each production stage, the data has been normalized to the zero-degree position (cell area directly facing the light source).

It was observed that the soldered solar cells, solar E-filaments, and solar E-yarns performed best when the face of the solar cell was directly exposed to the light source, which would be expected. Once the cell was encapsulated, the clear resin micro-pod would act as a lens and would both capture and trap light within its structure. This encapsulation artificially increased the area over which the cell could capture light (it was no longer just capturing light over a flat surface but a larger curved surface). Hence, superior performance at a wider range of angles would be expected compared to a soldered solar cell, and this was observed when the cell was rotated in the clockwise direction. The inclusion of the fibrous braid further reduced the sensitivity of the solar E-yarn to the angle of the incident light. This may be due to the scattering of the light within the braid’s structure. Any change in the sensitivity to the incident angle was only seen when the sample was rotated ‘clockwise’. This was believed to be due to the presence of the soldered Litz wire on one side of the cell. At increasing angles in one direction the Litz wire soldered onto the front contact of the cell would increasingly block the light reaching the cell, which would be most evident when the soldered solar cell was tested. The effects of the encapsulation and braid, therefore, had a noticeable effect on the amount of light captured under these conditions.

### Appendix A.3. Demonstration of the Deformability of the Large Woven Textile Solar Panel

While textile solar panels of this type have previously been demonstrated to be highly deformable [1], Figure A3 shows the large area woven textile solar panel being mechanically deformed as further evidence of its pliable nature.
